# Imaging characteristics of orbital tumors: A case series analysis

**DOI:** 10.6026/9732063002001299

**Published:** 2024-10-31

**Authors:** Sachin Dawale, Naresh Vishwanath Iyer Murali, Ruchi Kothari, Nishanth Gejjalagere Chandrashekar, Mayur Wanjari, Ravi Sangoi, Krisha Jain

**Affiliations:** 1 Department of Radiodiagnosis, Mahatma Gandhi Institute of Medical Sciences, Sevagram, Wardha, India; 2 Department of General Medicine, United Lincolnshire Hospitals NHS Trust, United Kingdom; 3 Department of Physiology, Mahatma Gandhi Institute of Medical Sciences, Sevagram, Wardha, India; 4 Department of General Medicine, United Lincolnshire Hospitals NHS Trust, United Kingdom; 5 Department of Research, Datta Meghe Institute of Higher Education & Research (DMIHER), Sawangi, Maharashtra, India; 6 Department of Internal Medicine, Government Medical College, Baramati, India

**Keywords:** Brain tumors, visual evoked potentials, magnetic resonance imaging, intra-orbital, orbital tumors

## Abstract

Orbital tumors are a diagnostic and therapeutic challenge due to their varied etiologies and potential for severe complications, either of vision and systemic status. This case series particularly highlights the clinical presentation, imaging features and outcomes of management of three patients with different types of orbital masse. This includes lacrimal gland neoplasm, choroidal melanoma with metastasis and intra-conal mass suspicious of intra-orbital melanoma.

## Background:

Ocular manifestations of systemic diseases often remain undiagnosed and play a critical role in the management of patients. Orbital masses are a diagnostic challenge and relatively rare. They can occur for several etiologies, including benign inflammatory lesions or malignant tumors [1]. These masses can have potentially profound impacts on vision and quality of life; thus, early diagnosis and treatment are paramount [2]. Advanced imaging techniques, like Magnetic Resonance Imaging, have an important role in the characterization and management of these lesions [3]. Differentiation between benign and malignant diseases is very much considered important issues in managing orbital masses, as therapeutic methods differ so widely between the two. The critical distinction is made because the disease might have to be treated promptly-thus a difference in treatment means a difference in survival rate. For instance, choroidal melanoma is the most common primary intraocular malignancy in adults and metastasizes if not appropriately managed [4]. Similar examples are lacrimal gland tumors, that must be differentiated carefully with the benign pleomorphic adenomas and malignant adenoid cystic carcinomas because, similar to the latter, they entail poor prognosis owing to their aggressive behavior and the possibility of peri-neural invasion [5].

## Case 1:

73 years old male patient came to the eye OPD with chief complaint of diminution of vision in the right eye (RE) since 9 months. On examination visual acuity (VA) RE finger counting close to face (FCCF) Projections of rays (PR) INACCURATE LE 6/12 PHI 6/9 PR ACCURATE, Intra-Ocular Pressure (IOP) RE 14.6 mm Hg LE 14.6 mm Hg Blood Pressure (BP) -110/80 mmHg, Random Blood sugar (RBS) -140 mg/dl, Hirschberg corneal reflex (HBT) central, head posture maintained, eyeball RE within normal limits (WNL) LE WNL, eyelid RE WNL LE WNL, lacrimal apparatus RE regurgitation on pressure over the lacrimal sac (ROPLAS) negative LE ROPLAS negative, conjunctiva RE cicatrizing conjunctivitis (CC) present (+) sentinel vessel+ LE WNL, sclera RE WNL LE scar mark present, cornea RE clear and transparent LE clear and transparent, anterior chamber RE normal depth and contents LE normal depth and contents, iris RE WNL LE WNL pupil RE single circular 2- 3 mm, Relative Afferent Pupillary Defect, (RAPD) grade II, LE single circular 2-3 mm, direct- normally reacting to light Lens RE grey LE grey, fundus RE media clear disc hyperemic, normal shape, margin ill defined, cup to disc ratio cannot be assessed, arteriolar/venular ratio (A:V) 1.5:3, arteriolar attenuation present, background normal FR dull, LE media clear disc normal size, colour, shape, margin well defined ,cup to disc ratio 0.3:1, A:V 1.5:3, arteriolar attenuation present, background normal Foveal reflex (FR) present.

T1 & T2 WI were taken in sagittal, axial & coronal planes along with FLAIR axial & DWI images on a 1.5 Tesla Siemens MRI machine. Contrast study was done after written and informed consent. A well circumscribed right orbital extra conal mass lesion of approximate size: 2.6 x 2.5 x 2.0 cm occupying the right lacrimal fossa and involving & compressing the right lateral rectus inferno-medially, showing diffusion restriction on DWI/ADC imaging and the pushing the globe anteriorly. The lesion appears isointense on T1, T2 and STIR with uniform enhancement on post contrast study. Left lacrimal gland appears bulky showing diffusion restriction & post contrast uniform enhancement. Left orbit show normal shape, size, symmetry with smooth borders. Left globe show normal MR morphology and position. Bilateral optic nerves show normal course, caliber and normal signal intensity. No thickening or abnormal contrast enhancement noted in the optic nerves.

## Optic nerve dimensions:

Orbit/segment Retrobulbar Right 5.0 mm Left 5.0 mm (Normal range: 5.5 +/-0.8 mm) Mid-orbit (narrowest) Right 4.0 mm Left 4.2 mm (Normal range: 4.2 +/-0.6 mm).

MRI Orbit reveals Right lacrimal gland extraconal space occupying lesion involving right lateral rectus muscle. Left lacrimal gland is bulky showing diffusion restriction. Findings may suggest bilateral lacrimal gland neoplastic lesion.

## VEP Findings (Figure 1 - see PDF):

PRVEP study shows markedly reduced P100 Amplitude and P100 latency WNL in Rt. Eye recording. P100 Latency and amplitude is WNL in Lt. Eye recording.

## Case 2:

## Right Eye (RE):

Choroidal Melanoma with Exudative Retinal Detachment, Nuclear Sclerosis II with Cortical Cataract with Lenticular Myopia

## Left Eye (LE):

Nuclear Sclerosis with Cortical Cataract with Lenticular Myopia with Atrophic Nasal Pterygium Grade I with Brain and Lung Metastasis 72-year-old male patient came to the eye OPD with a chief complaint of blurring of vision for both near and distant in the right eye for 2 months. On examination VA RE FC 2M PH NO IMPROVEMENT PR INACCURATE LE 6/18 PHI 6/12 PR SYR RE FP LE FP BP -130/80 mmhg RBS -143 mg/dl, HBT central, head posture maintained, eyeball RE WNL LE WNL, eyelid RE WNL LE WNL, A lacrimal gland RE ROPLAS negative LE ROPLAS negative, conjunctiva RE CC+ sentinel vessels+ LE WNL, sclera RE WNL LE WNL, cornea RE clear and transparent LE clear and transparent, anterior chamber RE normal depth and content LE normal depth and content, iris RE WNL LE WNL pupil RE single circular 2-3 mm, RAPD, LE single circular 2-3 mm, normally reacting to light Lens RE grey LE amber, fundus RE media clear disc hyperemic distorted shape, margin ill defined, cup to disc ratio could not be assessed, A: V 2:3, background 2-3 disc diameter uveal mass supero nasal temporal disc with exudative retinal detachment surrounding FR dull, LE media hazy disc normal size, colour, shape, margin well defined, cup to disc ratio 0.3:1, A:V 2:3, background WNL FR present. T1 & T2 WI were taken in sagittal, axial & coronal planes along with FLAIR axial & DWI images on a 1.5 Tesla Siemens MRI machine. Contrast study was done after written and informed consent.

## Findings:

Well-defined intraocular heterogeneously mildly enhancing altered intensity lesion, of approximate size 1.3 x 1.3 x 0.5 cm (CC x AP x TR), noted along the postero- medial aspect of right eye, appearing hyperintense on T1W/FLAIR & hypointense on T2W sequences, showing no diffusion restriction on DWI/ADC or blooming on SWI/GRE sequences. Left orbit shows normal shape, size and symmetry with smooth borders. Bilateral globes show normal MR morphology and position. Left optic nerve shows normal course, caliber and normal signal intensity. No thickening or abnormal contrast enhancement noted in the left optic nerve.

## Optic nerve dimensions:

Orbit/segment Right Left Normal Retrobulbar 5.1 mm 5.6 mm 5.5 +/-0.8 mm Mid-orbit (narrowest) 4.0 mm 4.3 mm 4.2 +/-0.6 mm.

## Impression:

MRI Orbit reveals - Well-defined intraocular heterogeneously mildly enhancing altered intensity lesion, along the postero- medial aspect of right eye, likely suggestive of uveal malignant melanoma.

## Technique:

T1 & T2 WI were taken in sagittal, axial & coronal planes along with FLAIR axial & DWI images on a 1.5 tesla Siemens MRI machine. Contrast study was done after written and informed consent.

## Findings:

Multiple well-defined round to oval variable sized enhancing altered signal intensity lesions in bilateral cerebral 2 cerebellar hemispheres, largest of approximate size 1.5 X 1.3 cm, appearing isointense on T1W/T2W/FLAIR sequences, showing no diffusion restriction on DWI/ADC Or blooming on SWI/ GRE sequences. No peri - lesional edema noted. Few punctate white matter hyperintensities in bilateral periventricular deep white matter and bilateral centrum semiovale. Prominent perivascular spaces noted in bilateral basal ganglia. Bilateral basal ganglia, thalami, internal & external capsules show normal signal intensity. Mid brain, pons & medulla shows normal MR morphology & signal intensity. Ventricular system, basal cistern and sulco gyral spaces appear prominent. Major cerebral vessels show normal flow voids. Imaged PNS: Non-enhancing mucosal wall thickening of bilateral ethmoid & left maxillary sinuses, suggestive of Sinusitis: Imaged mastoids shows normal MR morphology & signal intensity.

## Impression:

MRI brain with contrast reveals - Multiple well-defined round to oval variable sized enhancing altered signal intensity lesions in bilateral cerebral & cerebellar hemispheres, likely suggestive of metastasis. Grade I fazekas lesions in bilateral periventricular deep white matter and bilateral centrum semiovale. Age related brain parenchymal changes.

## USG Orbit Findings:

## Right orbit:

## Axial length:

22.1 mm Evidence of well-defined hyperechoic lesion of approximate size 1.3x 0.5 cm arising from posterior orbital wall protruding in viterous chamber involving optic disc, showing vascularity on colour doppler imaging. Evidence of early cataractous changes noted in the lens. Evidence of partial retinal detachment and vitreous haemorrhage No evidence of vitreous degeneration and vitreous detachment and choroidal detachment. Rest of the optic nerve normal in caliber and course. Retro-orbital complex normal in morphology and echotexture.

## Left orbit:

## Axial length:

22.2 mm. Evidence of early cataractous changes noted in the lens. Evidence of vitreous degeneration and vitreous detachment. No evidence of retinal detachment and choroidal detachment. Optic nerve normal in caliber and course. Retro-orbital complex normal in morphology and echotexture.

## Impression:

## USG Orbit reveals:

## Right eye:

Well defined hyperechoic lesion arising from posterior orbital wall protruding in viterous chamber involving optic disc, likely suggestive of neoplastic etiology. Early cataract with partial retinal detachment, vitreous haemorrhage. Left eye: Early cataract with vitreous degeneration & detachment.

## VEP Findings (Figure 2 - see PDF):

PRVEP study shows Markedly Prolonged P100 Latency in Rt. Eye recording and P100 Latency is WNL in Lt. Eye recording. P100 Amplitude is reduced in RE recordings and WNL in LE recording.

## Case 3:

38 years old female patient came to the eye OPD with chief complaint of blurring of vision in the right eye since 8 months. On examination VA RE FCCF PH NO IMPROVEMENT PR INACCURATE LE 6/6 PR ACCURATE, IOP RE 14.6 mm Hg LE 14.6 mm Hg SYR RE FP LE FP BP -120/80 mmHg RBS -138 mg/dl, HBT central, head posture maintained, eyeball RE WNL LE WNL, eyelid RE WNL LE WNL, lacrimal apparatus RE ROPLAS negative LE ROPLAS negative, conjunctiva RE CC+ sentinel vessels+ LE WNL, sclera RE WNL LE WNL, cornea RE clear and transparent LE clear and transparent , anterior chamber RE normal depth and contents LE normal depth and contents, iris RE WNL LE WNL pupil RE single circular 2- 3 mm, RAPD grade II, LE single circular 2-3 mm, direct- normally reacting to light Lens RE grey LE grey, fundus RE media clear disc hyperemic distorted shape, margin ill defined, cup to disc ratio cannot be assessed, A: V 2:3, background 2-3 disc diameter uveal mass supero nasal to disc extending to macula FR dull, LE media clear disc normal size, colour, shape, margin well defined, cup to disc ratio 0.3:1, A:V 2:3, background normal FR present. T1 & T2 WI were taken in sagittal, axial & coronal planes along with FLAIR axial & DWI images on a 1.5 Tesla Siemens MRI machine. Contrast study was done after written and informed consent. Heterogeneously enhancing well defined lobulated altered signal intensity mass lesion of approximate size 2.7 x 2.0 x 1.4 cm noted in right orbit between the optic nerve and medial rectus. The mass is separate to, but displaces the optic nerve laterally. There is no fat plane between the mass and the medial rectus, but the belly of medial rectus is of normal caliber. The lesion is displacing adjacent retro-orbital fat laterally, displacing right globe anteriorly, posteriorly touching the orbital apex, appearing heterogeneously hyperintense to grey matter on T1W, heterogeneously hyperintense on T2W/STIR , diffusion restriction on DWI sequence and marked areas of blooming on SWI imaging.

[1] There is no obvious bony involvement or intra cranial involvement.

[2] Right cavernous sinus appear normal

[3] Left orbit shows normal shape, size, symmetry with smooth borders

[4] Left globe shows normal MR morphology and position.

[5] Left optic nerve show normal course, caliber and normal signal intensity.

[6] No thickening or abnormal contrast enhancement noted in the optic nerves.

## Optic nerve dimensions:

Orbit/segment Retrobulbar Right 5.0 mm Left 5.2 mm (Normal range: 5.5 +/-0.8 mm) Mid-orbit (narrowest) Right 4.0 mm Left 4.3 mm (Normal range: 4.2 +/-0.6 mm).

## Impression:

## MRI Orbit may suggest:

Enhancing lobulated right orbital intraconal extraocular mass lesion with extensions and involvements suggestive of neoplastic etiology? Intraorbital melanoma? Optic nerve sheath Meningioma.

## VEP Findings (Figure 3 - see PDF):

PRVEP study shows no absolute P100 waveform in Rt. Eye recording. P100 Latency and amplitude is WNL in Lt. Eye recording.

## Discussion:

One of the major findings in this study is benign lesions, which would include dermoid cysts and pleomorphic adenomas and this corresponds to some literature findings that indicate most orbital tumors in adults are benign [3]. Detection of the ominous cases such as choroidal melanoma and adenoid cystic carcinoma of the lacrimal gland essentially calls for great alertness in clinical assessment and the use of imaging in early diagnosis and intervention [4, 5]. These findings have important clinical implications. The timely and accurate differentiation of orbital masses can prevent unnecessary invasive procedures in a timely fashion and allow the initiation of appropriate therapies to ameliorate both visual and systemic outcomes [6]. Additionally, knowledge of the typical imaging characteristics of orbital tumors can aid in formulating differential diagnoses, thus introducing more personal and effective management approaches [7, 8]. Our study, despite these contributions, comes with several limitations. Analysis of this kind has always been retrospective and the sample size is relatively small. These may limit the generalizability of our findings. Larger cohorts and prospective designs will be necessary in future studies to validate these results further and better define the role that emerging imaging techniques, such as PET-CT and advanced MRI sequences, will play in the management of orbital tumors [9].

## Conclusion:

Data shows the important role of MRI in the diagnostic workup of orbital tumors and highlights the importance of a high index of suspicion in clinical practice. Further research into the pathophysiology and imaging characteristics of these lesions is necessary for maximizing accuracy and outcomes in diagnosis and patient care.
